# Supplementary material for: Voltammetry in the spleen assesses real-time immunomodulatory norepinephrine release elicited by autonomic neurostimulation
Source: J Neuroinflammation. 2023 Oct 17;20:236. doi: 10.1186/s12974-023-02902-x (PMC10583388; doi:10.1186/s12974-023-02902-x)
Supplement: Supplementary file 1 — Additional file 1: Figure S1. (a) Average oxidation potential (Eo) of NE in vitro (in PBS and in blood, 3.3 µg/mL, n = 3 in each condition) and in vivo (in live spleen, 0.5 µg/mL, n = 6). p by ANOVA with multiple comparisons. (b) Averaged and time-resolved voltammograms from experiments in 3 animals in each of which NE (approximately 100–200 µL of 10 µg/mL NE) or saline (equivalent volume) was administered as IV bolus; also shown are the respective io traces, for both NE and saline injections, demonstrating how the algorithm identifies temporal boundaries of the NE signal. (c) Qo values calculated using the temporal boundaries shown in (b). Figure S2. Left celiac–superior mesenteric (CSM) ganglion complex is identified using fluorescence microscopy in ChAT–tdTomato mice (ChAT+ tissue appears in red). The splanchnic nerve is isolated and cuffed with a bipolar stimulating electrode. [file 12974_2023_2902_MOESM1_ESM.docx]

**Additional file 1**


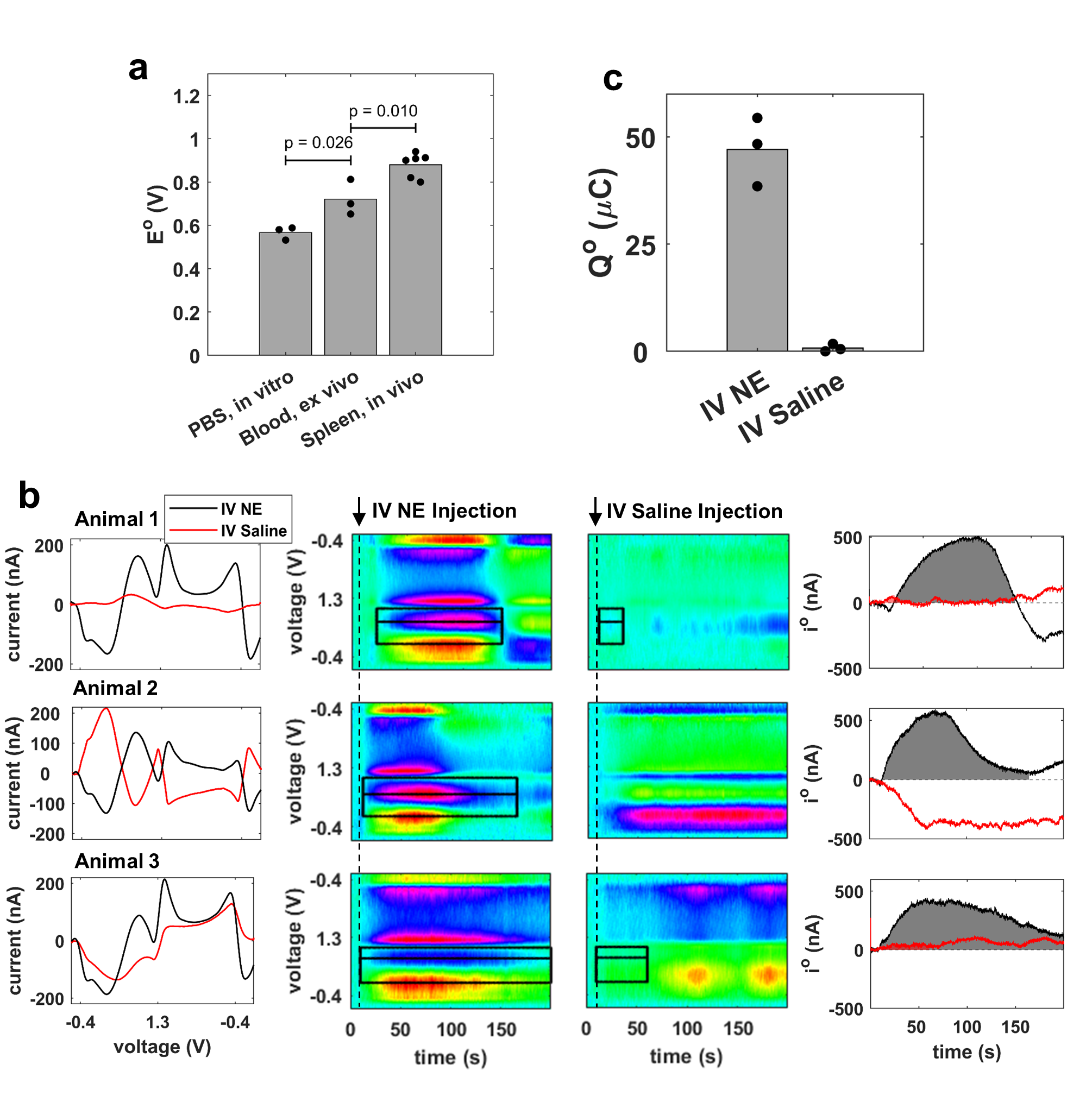


**Figure S1**. (a) Average oxidation potential (E^o^) of NE in vitro (in PBS and in blood, 3.3 µg/mL, n = 3 in each condition) and in vivo (in live spleen, 0.5 µg/mL, n = 6). *p* by ANOVA with multiple comparisons. (b) Averaged and time-resolved voltammograms from experiments in 3 animals in each of which NE (approximately 100-200 µL of 10 µg/mL NE) or saline (equivalent volume) was administered as IV bolus; also shown are the respective i^o^ traces, for both NE and saline injections, demonstrating how the algorithm identifies temporal boundaries of the NE signal. (c) Q^o^ values calculated using the temporal boundaries shown in (b).


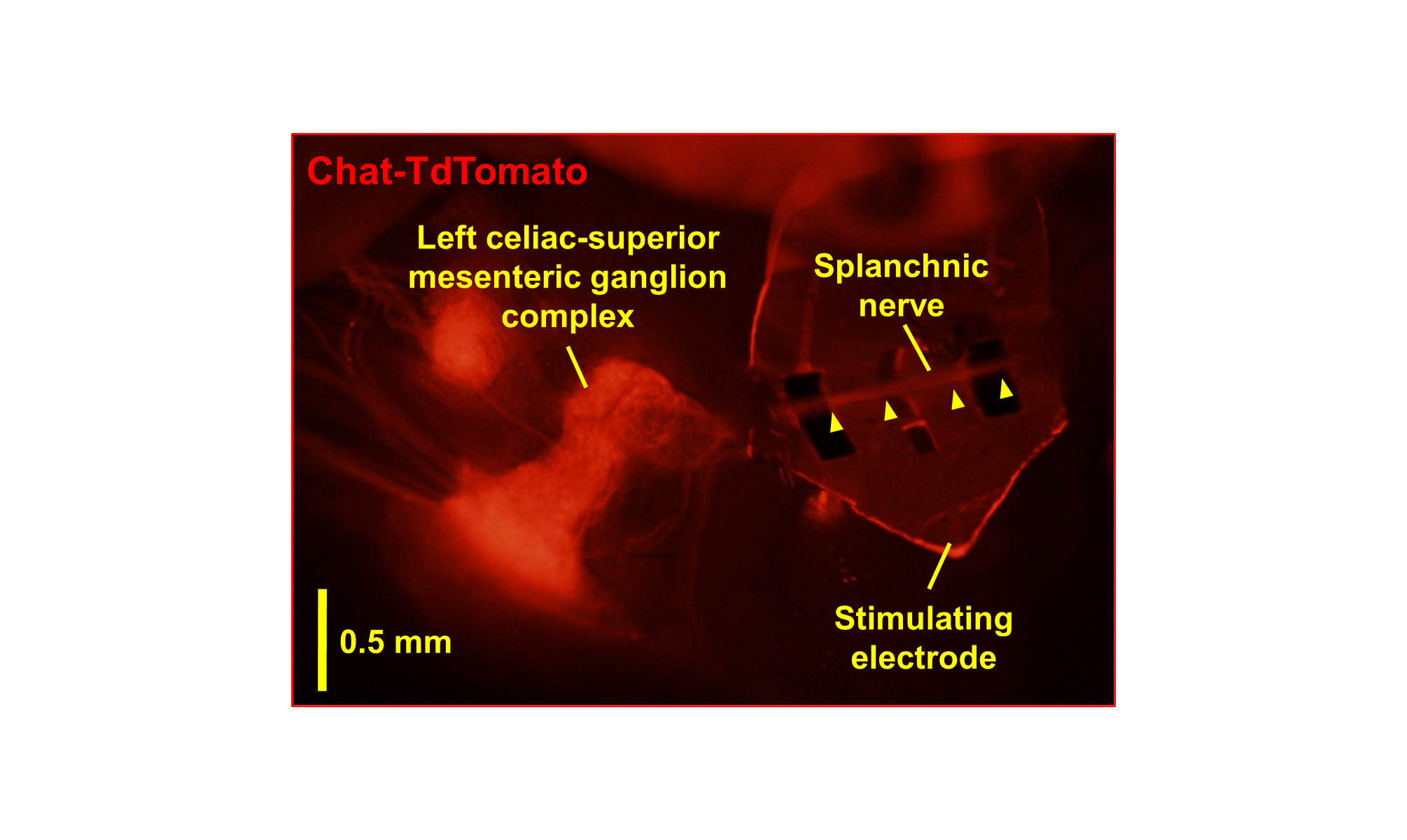


**Figure S2.** The left celiac-superior mesenteric (CSM) ganglion complex is identified using fluorescence microscopy in ChAT-tdTomato mice (ChAT^+^ tissue appears in red). The splanchnic nerve is isolated and cuffed with a bipolar stimulating electrode.

## NE Voltammetry Signal maintains its shape across stimulation conditions

Voltammetric measurements can be affected by changes in the immediate environment of the working electrode, including temperature, chemical and biological factors, electrode surface area, and pH (35). In our studies, we performed FSCV in a large number of animals over several months, while regularly replacing the working electrode; in addition, FSCV was performed in combination with a variety of autonomic stimulation conditions and parameters. When overlaid, averaged raw voltammograms from all stimulation conditions have qualitatively consistent shapes (Fig 4a). Values for NE oxidation potential were consistent among animals within same stimulation condition, and similar across different stimulation conditions (Fig. 4c). Peak oxidation currents ranged from 21.5 to 688.3 nA, while Q^o^ ranged from 2.3 to 121.7 µC across all modalities (Fig. 4b, d, & e). It is worth noting that even though VNS parameters were relatively lower than SpNS, VNS produced lower Q^o^ and peak i^o^ (Fig. 4d & e) despite causing significant drops in heart rate and changes in breathing.

# Supplementary Methods

## Algorithm for determining the integration window for i^o^

In order to integrate i^o^, two boundaries of integration need to be defined that ideally fit as closely as possible to a potential NE signal. The algorithm for identifying this signal is composed of 4 parts: a) identify the presence of the signal, b) identify presence of peaks based on identifying characteristics (minimum threshold and proximity to stimulus), c) remove peaks that are not bounded by discrete bounds, d) combine peak boundaries that meet criteria into final integral bounds.

For the 1st step, the NE signal had to be characterized so that it could be detected automatically. A standard voltammogram for NE was defined by averaging the resulting voltammograms from 5 venous NE injection experiments. This standard voltammogram was normalized and the dot product between itself and each voltammogram in the time series of an experiment was used to determine the presence, or absence, of the signal.

For the 2nd step, the dot product trace was processed using MATLAB’s peak detection algorithm with a minimum prominence threshold for detecting local peaks. The minimum prominence threshold was identified by calculating the prominence of the peaks generated from 0.02 µg of injected NE (Fig. 1c) from 3 experiments and the lowest was used as the threshold. Detected peaks were then restricted to those between the start of the stimulus and 1 minute after the end of the stimulus (experimentally determined from nerve stimulation studies).

For the 3rd step, peaks that were not bounded on either side by a valid bound were classified as invalid. A valid bound was defined as either a local minima (i.e. trough) or a zero value. In cases where both a minima and a zero were present, the zero was preferred. The start of the stimulus was also considered a valid bound and overwrote minima/zeros that preceded it.

For the 4th step, the bounds of the remaining peaks were amassed and a final set of boundaries were set by the lowest to the highest of valid bounds. These integration bounds were applied to the i^o^ trace processed through a moving average filter of size 10s to eliminate spurious peaks and electrical artifacts. If no valid bounds met criteria, the signal was considered sub-threshold and Q^o^ given a value of zero. Algorithm implemented in MATLAB with all of the raw data can be found here: <https://github.com/mgerber000/SpleenFSCV>
